# NiCo_2_O_4_/RGO Hybrid Nanostructures on Surface-Modified Ni Core for Flexible Wire-Shaped Supercapacitor

**DOI:** 10.3390/nano11040852

**Published:** 2021-03-26

**Authors:** Prashant Shivaji Shewale, Kwang-Seok Yun

**Affiliations:** Department of Electronic Engineering, Sogang University, Seoul 04107, Korea; prashantshewale11@gmail.com

**Keywords:** wire, flexible, supercapacitor, NiCo_2_O_4_, RGO

## Abstract

In this work, we report surface-modified nickel (Ni) wire/NiCo_2_O_4_/reduced graphene oxide (Ni/NCO/RGO) electrodes fabricated by a combination of facile solvothermal and hydrothermal deposition methods for wire-shaped supercapacitor application. The effect of Ni wire etching on the microstructural, surface morphological and electrochemical properties of Ni/NCO/RGO electrodes was investigated in detail. On account of the improved hybrid nanostructure and the synergistic effect between spinel-NiCo_2_O_4_ hollow microspheres and RGO nanoflakes, the electrode obtained from Ni wire etched for 10 min, i.e., Ni_10_/NCO/RGO exhibits the lowest initial equivalent resistance (1.68 Ω), and displays a good rate capability with a volumetric capacitance (2.64 F/cm^3^) and areal capacitance (25.3 mF/cm^2^). Additionally, the volumetric specific capacitance calculated by considering only active material volume was found to be as high as 253 F/cm^3^. It is revealed that the diffusion-controlled process related to faradaic volume processes (battery type) contributed significantly to the surface-controlled process of the Ni_10_/NCO/RGO electrode compared to other electrodes that led to the optimum electrochemical performance. Furthermore, the wire-shaped supercapacitor (WSC) was fabricated by assembling two optimum electrodes in-twisted structure with gel electrolyte and the device exhibited 10 μWh/cm^3^ (54 mWh/kg) energy density and 4.95 mW/cm^3^ (27 W/kg) power density at 200 μA. Finally, the repeatability, flexibility, and scalability of WSCs were successfully demonstrated at various device lengths and bending angles.

## 1. Introduction

As one kind of significant energy storage device, supercapacitors (SCs) can function at a high charge/discharge rate over a large number of cycles, covering the gap amongst high energy batteries and high power conventional electrostatic capacitors [1,2]. Due to their favorable characteristics, SCs are useful in hybrid vehicles, portable electronics, aviation equipment, and backup energy systems [3]. Commonly, SCs are fabricated in basic planar or two-dimensional (2D) configurations; however, they do not offer sufficient flexibility to satisfy the demands for future flexible wearable electronic devices and so cannot be simply woven into textiles. As a result, more recently, fiber type SC design, lightweight and with more flexibility has become popular [4,5,6,7]. The fiber-shaped or wire-shaped supercapacitors (FSCs or WSCs) provide machinability, breathability, and manufacturing flexibility to woven fabrics for application in textiles [8,9]. 

To achieve high-performance flexible SCs, many hybrid nanostructures with metal oxide and carbon-based materials have been studied. Among various metal oxide electrode materials, NiCo_2_O_4_ (NCO) is one of the most promising supercapacitor materials because of its high theoretical capacity [10,11], though the electrochemical performance of NCO-based flexible electrodes is restricted because of their low electrical conductivity. Thus, the methodology of combining faradaic materials like NCO with conductive carbonaceous materials [12,13,14] or conducting polymers [15,16] has been severally implemented for the enhancement of the electrochemical performance, as the conductive materials can increase electric conductivity and cycling stability. Particularly, the reduced graphene oxide (RGO) has been widely applied as a support for metal oxides in electrochemical electrode materials because of its large surface area, high conductivity, high chemical stability, and excellent mechanical flexibility [17,18,19,20]. Therefore, combining both these materials as hybrid nanostructures for the fabrication of the electrodes for SCs becomes indispensable given the electrochemical performance improvement due to their synergistic effects. 

Furthermore, as a current collector, metal wires have been seen perform a crucial role in obtaining the flexible SCs, predominantly owing to their superior mechanical strength and high electrical and thermal conductivity [21,22,23,24,25,26]. The application of a highly conductive core such as metal wire is considered to enhance the flexible SC performance by the reduction in loss of charge, and simultaneous improved transmission of electrical power and charge storage [27,28]. However, metal wires also possess major downsides such as the weak adhesion between the electrode and electroactive materials, low surface area, and low porosity. One of the most effective ways to get rid of such drawbacks is the direct growth of electrode materials with high porous structures on conductive substrates [24,25,26]. Additionally, the substrate roughening treatment has been recognized to improve the film adhesion to the substrate [29] and it has also been seen to increase the surface area, thus providing more reactive sites for surface modification [30,31]. Therefore, substrate surface roughening can be an effective strategy in extending the cycling life of the electrode and its energy storage capacity [29]. Although considerable research has been conducted into flexible SCs, to the best of our knowledge, hitherto there are no reports on the enhancement of electrochemical performance of symmetric WSCs with electrodes fabricated by facile direct solvothermal and hydrothermal growth of electroactive NiCo_2_O_4_/RGO hybrid nanostructures onto surface modified Ni wires as a current collector. 

Therefore, herein, we report the successful fabrication of high-performance flexible twisted WSC based on Ni wires coated with NCO microspheres and RGO nanoflakes using simple and inexpensive direct solvothermal and hydrothermal growth methods, respectively. Flexible Ni/NCO/RGO electrodes have been prepared by roughening the surface of Ni wire current collectors by a chemical etching process over various time durations and their electrochemical properties have been systematically evaluated to construct the WSC. The Ni wire surface roughness treatment resulted in noteworthy modifications to the electrode’s surface morphology and hence its capacitive properties. Owing to the highly porous hybrid nanostructure and the synergistic effects between NCO hollow microspheres and RGO nanoflakes, the WSC based on two twisted Ni_10_/NCO/RGO electrodes exhibits enhanced electrochemical properties. Furthermore, the device shows highly stable electrochemical characteristics under different deformations such as bending and winding.

## 2. Experimental

### 2.1. Preparation of Wire Electrodes

Before the synthesis of the hybrid nanostructures, the surface of the Ni wire (diameter: 300 μm) was thoroughly cleaned using acetone, ethanol, and deionized (DI) water. Then the precleaned surface of the Ni wires was modified by wet etching with an etchant solution made up of hydrochloric acid (HCl, Sigma Adrich, St. Louis, MO, USA) and nitric acid (HNO_3_, Sigma Adrich, St. Louis, MO, USA) in the ratio of 4:1 for 10, 20, and 30 min. Thereafter, to synthesize NCO microspheres on the etched Ni wire surface, we employed a facile solvothermal process. Typically, to prepare hollow NiCo_2_O_4_ microspheres onto Ni wire surface, cobalt nitrate (Co(NO_3_)_2_·4H_2_O, Sigma Adrich, St. Louis, MO, USA) (1.74 g), nickel nitrate (Ni(NO_3_)_2_·6H_2_O, Sigma Adrich, St. Louis, MO, USA) (0.87 g), and urea (CH_4_N_2_O, Sigma Adrich, St. Louis, MO, USA) (10.8 g) was dissolved in a mixture of 45 mL isopropyl alcohol (IPA, Sigma Adrich, St. Louis, MO, USA) and 9 mL DI water. The resulting solution was continuously stirred for 1 h and then transferred into a Teflon-lined stainless-steel autoclave containing unetched/etched Ni wires, which was then placed in an electric oven and heated at 120 °C for 12 h. After completion of this solvothermal reaction, the autoclave was naturally cooled down to room temperature. The gray-colored precursor coated Ni wires were collected, cleaned with DI water and naturally dried. Lastly, the dried as-synthesized Ni wires were calcined at 350 °C for 2 h to obtain black colored wires coated with hollow NCO microspheres. Further, these wires were immersed in a homogeneous aqueous solution of graphene oxide (GO) and urea contained in an autoclave vessel, and a hydrothermal reaction was carried out at 120 °C for 8 h to obtain RGO coated Ni/NCO wires which were again calcined at 350 °C for 2 h. For convenience, the electrodes obtained with unetched and 10, 20, 30 min etched Ni wires were denoted as Ni_00_/NCO/RGO, Ni_10_/NCO/RGO, Ni_20_/NCO/RGO, and Ni_30_/NCO/RGO, respectively.

### 2.2. Fabrication of Wire-Shaped Symmetric Supercapacitor

To construct FSCs using the unetched and etched Ni wires with NCO/RGO hybrid nanostructures, firstly an electrolyte was prepared by mixing 6 g of potassium hydroxide (KOH, Sigma Adrich, St. Louis, MO, USA), 10 g of polyvinyl alcohol (PVA, Sigma Adrich, St. Louis, MO, USA), and 100 mL DI water, which was heated at 80 °C with a constant stirring until a viscous gel was formed. Thereafter, the prepared electrolyte was uniformly coated onto Ni/NCO/RGO electrodes using the dip-coating method and one end of these electrodes was polished to form current collectors. Then, two electrodes with their electrolyte-coated portions were closely twisted together to form the twisted-type WSC. 

### 2.3. Materials Characterization

The structural, morphological, and compositional characterization of prepared hybrid nanostructures were examined by using X-ray diffraction (XRD) and field-emission scanning electron microscopy (FE-SEM, JSM-7100F, Tokyo, Japan) equipped with energy-dispersive X-ray spectroscopy (EDS). The XRD (Rigaku D/max-rA, Tokyo, Japan) with Cu-K_α1_ radiations (*λ* = 1.5406 Å) in the 2θ range from 10 to 80 (operate data voltage of 30 kV and current of 15 mA) was used to investigate crystallinity, phase formation, and the crystallite size of NCO/RGO active materials on each type of electrode. The surface morphology was assessed using an FE-SEM (JEOL, Tokyo, Japan); while the elemental compositions of the nanomaterials were determined by obtaining the EDS spectra and EDS mappings of electrodes. To further examine the electrode microstructure, transmission electron microscopy (TEM) (JEOL, JEM-F200, Tokyo, Japan) was used. Selected area electron diffraction (SAED) patterns and TEM images were recorded from the smallest components of the optimum electrode. Sample for TEM was prepared by dropping a dilute suspension of the active material sample in isopropanol onto a carbon-coated copper grid followed by drying under reduced pressure for 24 h before TEM observation. Mercury intrusion porosimetry (MIP) (Poremaster, Quantachrome Instruments, Boynton Beach, FL, USA) was used to detect the pore size distribution and the total pore volume of the active materials on each wire electrode in the macropore region. 

### 2.4. Electrochemical Characterization

For electrochemical characterization of fabricated single wire electrodes, cyclic voltammetry (CV), galvanostatic charge/discharge (GCD) tests, and electrochemical impedance spectroscopy (EIS) were conducted at room temperature with a potentiostat (VersaSTAT 300, Princeton Applied Research, Oak Ridge, TN, USA) in a three-electrode cell configuration with 2 M KOH electrolyte, an Ag/AgCl (in 3 M KCl) reference electrode and platinum (Pt) wire as a counter electrode. Finally, the electrochemical performance of the WSC fabricated with optimum electrodes was evaluated in a two-electrode configuration, where one end of the WSC is connected as a working electrode, while its opposite end is attached as reference and counter electrode. The photographs of the experimental setup for the single electrode characterization in the three-electrode configuration and WSC characterization in the two-electrode configuration are shown in Figure S1a,b, respectively. 

In the present study, the specific capacity *C*_sc_ (mAh/g) of electrodes and the supercapacitor was calculated from CV curves by using formula *C*_sc_ =∫*idt*/(3600*m*), where *m* is the mass loading of active material (g), and *i* is current over time [32]. The specific capacity of the electrodes, *Q_s_* (C/g) were further evaluated from the GCD curve using the relation as *Q_s_* = *I*Δ*t*/m [33]. Further, GCD curves were used to calculate the volumetric specific capacitance (*C_v_*), areal specific capacitance (*C_A_*), and length specific capacitance (*C_L_*) by using the formula *C_v_* = *I*Δ*t* (*v*Δ*V*), *C_A_* = *I*Δ*t*/(*A*Δ*V*) and *C_L_* = *I*Δ*t*/(*L*Δ*V*), respectively. Then, the energy density, *E* (Wh/cm^3^) and power density *P* (W/cm^3^) were calculated by the equations *E* = *C_v_*Δ*V*^2^ /7200 and *P* = 3600*E*/Δ*t* [34]. The energy and power densities in the dimensions of Wh/kg and W/kg, respectively, were also evaluated by using the formulae E = *C_sc_*Δ*V/*7.2 and *P* = 3600E/Δ*t* [33]. In all these equations, Δ*V* is the potential window, *I* is the discharge current, and Δ*t* is the discharge time, while *v*, *A* and *L* are the volume, surface area, and length of the electrode, respectively. 

## 3. Results and Discussion

### 3.1. Structural, Surface-Morphological and Chemical Compositional Analysis

**Figure 1:**
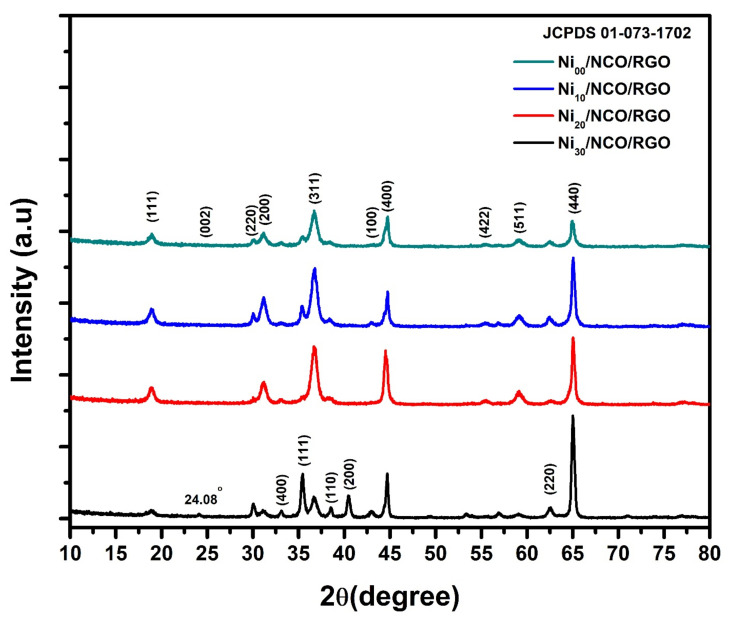
X-ray diffraction (XRD) patterns of various Ni/NCO/RGO hybrid nanostructures with different substrate etching durations.

To determine the synthesis of nano-crystallites in their pure phase and the formation of electroactive materials in the study, X-ray-diffraction (XRD) analysis was investigated. Figure 1 shows XRD patterns of Ni_00_/NCO/RGO, Ni_10_/NCO/RGO, Ni_20_/NCO/RGO, and Ni_30_/NCO/RGO electrodes. Similar kinds of diffraction peaks can be observed from the obtained XRD patterns for all electrodes. The XRD pattern of each electrode exhibits X-ray diffraction peaks at 19.0°, 31.4°, 30.06°, 36.9°, 44.9°, 55.56°, 59.6°, and 65.5° and all these peaks are well matching to the (111), (220), (200), (311), (400), (422), (511) and (440) planes of the cubic spinel-type structure of NiCo_2_O_4,_ consistent with the earlier reported literature and JCPDS 01-073-1702. Moreover, some extra XRD peaks at 33.1°, 35.46°, 38.52°, 40.44°, and 62.66° are also observed which seems to be associated with the NiO phase impurities, especially for unetched Ni/NCO/RGO electrode. After etching these NiO-related peaks almost disappeared, or their intensity was decreased. Further, the very low-intensity peaks at 24.08° and ~43.00° are also observed in all XRD patterns, confirming the existence of reduced graphene oxide that forms the hybrid nanostructure with NiCo_2_O_4_. The observed shift in RGO XRD peak from its usual position at ~26° to a lower angle at ~24° was considered to be due to the short-range order in stacked stacks [35]. The nonexistence of graphene oxide-related peak certifies to the complete reduction of the GO during the hydrothermal deposition of RGO using the GO aqueous solution. Further, Scherer’s formula was used to evaluate the crystallite size for each of the electrodes and it is observed that the crystallite size decreases from ~23 nm to ~18 nm with an increase in the Ni wire etching time. This indicates the gradual lowering of the NiCo_2_O_4_ crystallinity with increasing roughness of Ni wire. 

**Figure 2:**
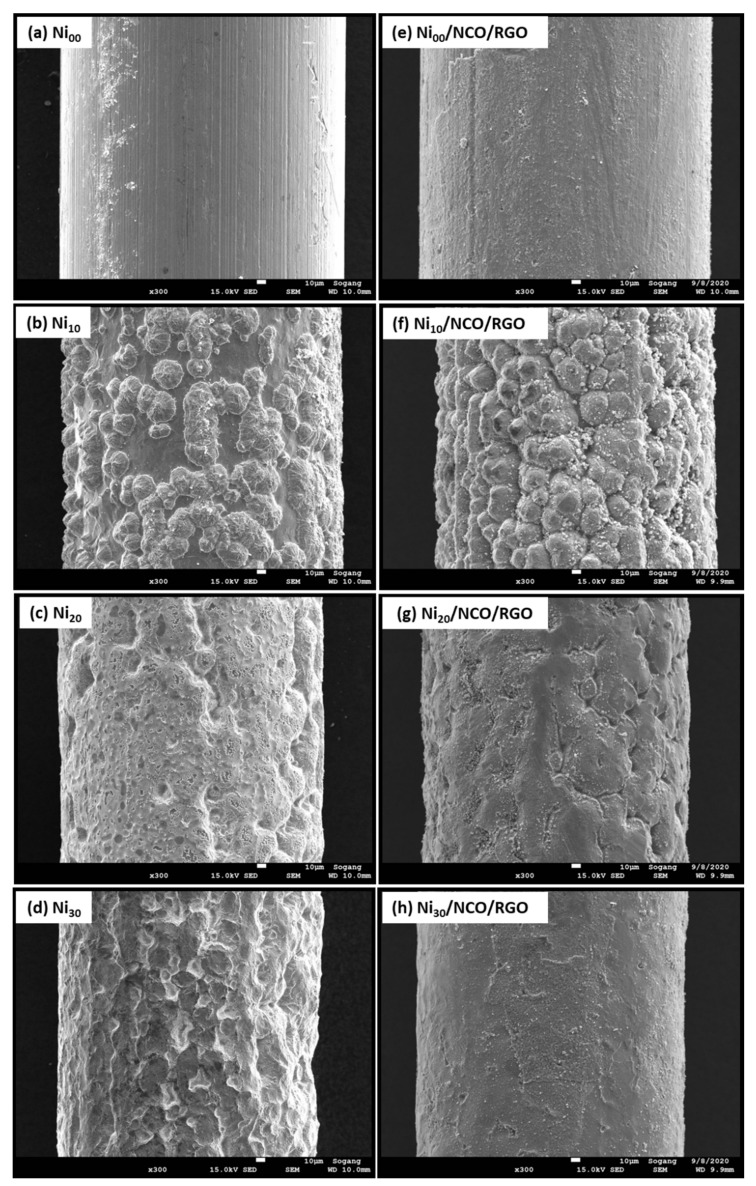
Field-emission scanning electron microscopy (FE-SEM) images of (**a**–**d**) Ni wires at different substrate etching durations, and (**e**–**h**) various Ni/NCO/RGO single electrodes.

**Figure 3:**
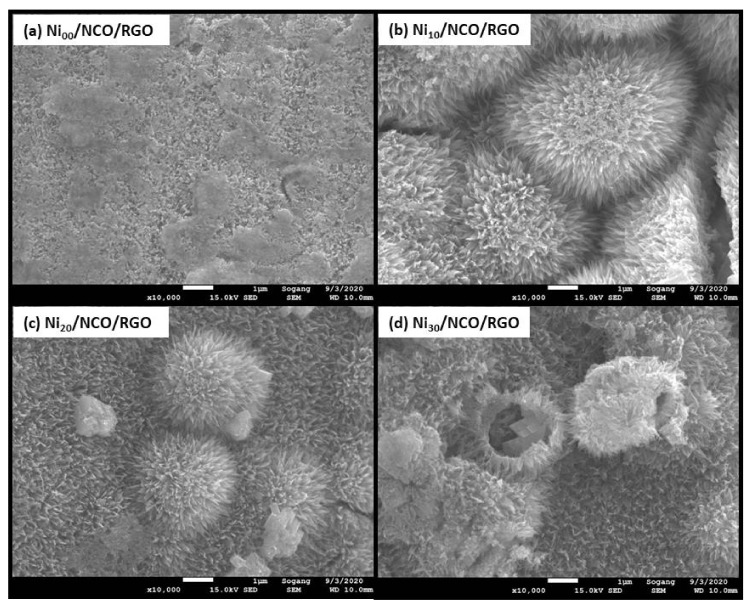
FE-SEM images of NCO/RGO hybrid nanostructures coated onto unetched and etched Ni wires.

Figure 2a–d shows the surface morphology of unetched and etched Ni wires. The effect of etching was observed to be most pronounced after 10 min of etching time with a larger roughened wire surface as compared to the relatively smoother surface of non-etched Ni wire. Such increased roughness is beneficial to increase film adhesion to the substrate, since the interfacial contact area between the substrate and the metal oxide coating increased. With a higher etching time than 10 min, more and more wire surfaces are etched-out making the wire surface smoother. The variation in Ni wire surface roughness has been seen to modify the surface morphology and hence the effective surface area of NCO/RGO hybrid nanostructures as shown in Figure 2e–h and Figure 3. Figure 2e–h shows the surface morphology of NCO/RGO hybrid nanostructures with coated unetched and etched Ni wires, where one can see very smooth surfaces for the Ni_00_/NCO/RGO and Ni_30_/NCO/RGO electrodes. The surface morphology of the NCO/RGO hybrid nanostructures, especially grown onto 10 min etched Ni wire, is very prominent, reflecting the obvious effect of the rough surface structure of the Ni wire. The high magnification FE-SEM images of all wire electrodes shown in Figure 3 imply a much clearer NCO/RGO surface morphological change driven by the Ni wire surface etching treatment. As seen from Figure 3a, the growth of microspheres is limited on Ni_00_ wire surface, hence resulting in relatively smooth surface morphology, whereas the proper roughened surface of Ni_10_ wire has resulted in substantial growth of the microspheres (Figure 3b), having numerous fine nanoneedles radially grown on their surfaces and clear boundaries between microspheres, though the size and density of the microspheres decreased on Ni_20_ and Ni_30_ wire surfaces (Figure 3c,d) with diminution in surface nanoneedles length. A broken microsphere observed in Figure 3d further confirms that the grown microspheres are hollow inside. Moreover, the number of adsorbed molecules or nucleation seeds on rough substrates surface was seen to considerably increase as compared with flat surfaces [36,37,38]. The rough surface offers an extra area to accommodate the nuclei. Besides, the rough surface could change the apparent contact angle between the crystal and the substrate, which would result in changing the energy for forming nuclei on the fractal surface, and thus influencing the heterogeneous nucleation process. In the present work, as 10 min of etching time has led to the highly roughened surface compared to the non-etched Ni wire, it provides an additional area to accommodate the nuclei, and thus exhibit a greater size and density of the microspheres; whereas, with a decreased surface roughness of the Ni_20_ and Ni_30_ wires, the availability of somewhat fewer nucleation seeds on their surface leads growth of relatively smaller and less dense microspheres. 

Based on the above observations, a possible mechanism of such hierarchical hollow microsphere formation could be similar to the one given by Yu et al. [39]. Firstly, the reaction of metal cations (Co^2+^ and Ni^2+^) with CO_3_^2−^ and OH^−^ anions gradually released from the hydrolysis of urea in an aqueous solution leads to the formation of cobalt-nickel bimetallic carbonate hydroxide nanoparticles, which are then gathered together into solid microspheres comprising flake like subunits. Then these microspheres experienced the first inside-out Ostwald-ripening and recrystallization process, and simultaneously nanoneedles started to develop on the surface of the nanoflakes. With further increase in reaction time, the formation of a yolk-shelled structure took place upon the second inside-out Ostwald-ripening and recrystallization process. As the reaction was further sustained, the microsphere hollowing course continued until totally hollow NCO microsphere precursors with well-defined hierarchical structures were obtained. 

The observed porous three-dimensional hollow microspheres of Ni_10_/NCO/RGO electrodes with high specific surface area would greatly improve the effective contact area for electrolyte ions at the electrode–electrolyte interface, which reduces the ion-diffusion path and enables the rapid redox reaction [40]. Figure S2 shows the corresponding EDS profiles of all the electrodes, and they confirm the presence of Ni, Co, O, and C elements in films. There are no obvious traces of any impurity in all hybrid nanostructures. The Pt signal evolved from conducting the coating carried out during FE-SEM and EDS analysis. These results further verify a successful synthesis of NCO/RGO hybrid nanostructures. The homogenous distribution of all four elements of Ni, Co, O, and C was further confirmed by obtaining the EDS elemental mapping images (Figure S3), where the C signal originates from the RGO nanoflakes onto the NCO microspheres, thus supporting the XRD patterns. 

**Figure 4:**
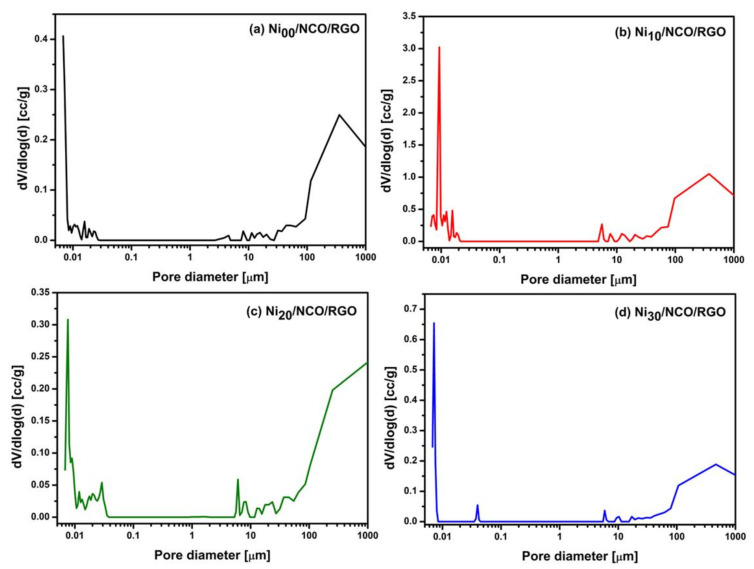
Pore size distribution curves of various Ni/NCO/RGO electrodes.

Various studies have shown that surface areas along with pore size distribution and percentage porosity all determine the ultimate performance of supercapacitors. Figure 4 shows the differential pore size distribution curves of various Ni/NCO/RGO electrodes. Two main characteristic pore ranges can be considered, small pores ranging from 0.006 to 0.1 μm and large pores over 5 μm. All the electrodes have differential peaks at pore diameters in the range of 0.006 to 0.1 μm. Nevertheless, as compared to other electrodes, the Ni_10_/NCO/RGO electrode exhibits the highest meso-porosity with pore diameters in the range of 6 to 22 nm. This electrode also exhibits greater macro-porosity compared to other electrodes. Such macropores resulting from electrode roughness ensure the wetting of the electrode surface and fast ion diffusion at higher applied current, therefore ensuring a high rate performance [41]. Further, the evident micropores are highly significant for enhancing ion diffusion kinetics that improves the capacitance behavior of supercapacitors while contributing to the lower equivalent resistance [42]. Moreover, the total surface area and total percentage porosity of Ni_00_/NCO/RGO, Ni_10_/NCO/RGO, Ni_20_/NCO/RGO, and Ni_30_/NCO/RGO electrodes varies at ~17, ~92, ~16, ~16 m^2^/g and ~79, ~98, ~71, and ~70%, respectively. Hence, Ni_10_/NCO/RGO with its superior surface area and porous nanostructure may exhibit better electrochemical properties, which is consistent with the morphological observations of FE-SEM.

**Figure 5:**
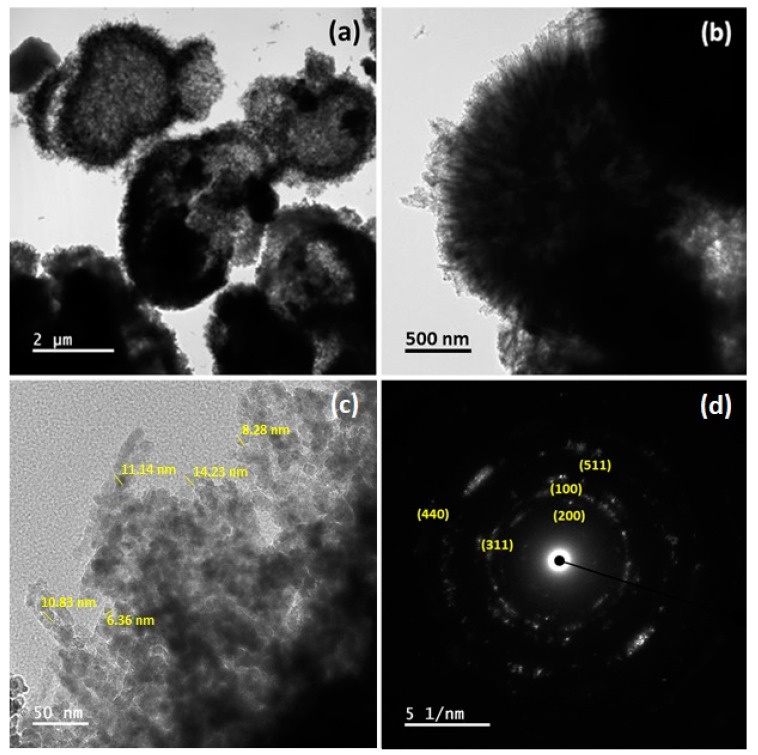
(**a**–**c**) Transmission electron microscopy (TEM) images at different magnifications and (**d**) selected area electron diffraction (SAED) pattern of Ni_10_/NCO/RGO hybrid nanostructures.

The microstructure and crystallographic properties of Ni_10_/NCO/RGO electrodes were further investigated thoroughly by using TEM and SAED. Microspheres of the as-synthesized electroactive material with outwardly grown nanoneedles on their surfaces are revealed by the TEM images in Figure 5a,b, which is consistent with the FE-SEM observations. It is also obvious from the high magnification TEM image in Figure 5c that these overgrown nanoneedles are composed of numerous nanoparticles with an average size of ~10 nm. The visibly blank spaces on some broken microspheres in Figure 5a and in between the nanoparticles in Figure 5c exemplify the formation of the mesoporous surface morphology. The SAED pattern of the electroactive material further ensured that as-synthesized material has a polycrystalline nature with well-defined rings as shown in Figure 5d. The identified ring pattern is well-indexed with characteristic planes of NCO and RGO, which is in line with the XRD results. 

### 3.2. The Electrochemical Characteristics of Ni/NCO/RGO Wire Electrodes

**Figure 6:**
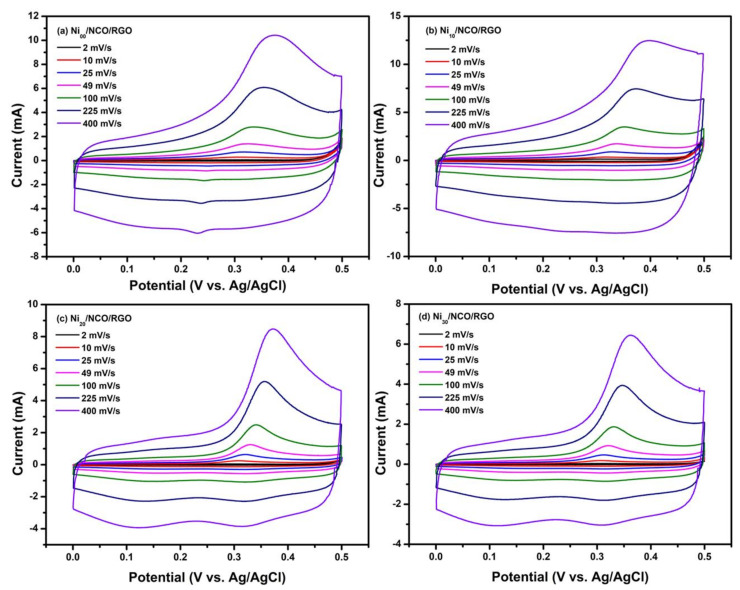
Cyclic voltammetry CV curves of different Ni/NCO/RGO electrodes at various scan rates under 0–0.5 V potential window.

The electrochemical performances of all Ni/NCO/RGO electrodes were measured by CV and GCD tests in 2.0 M KOH aqueous electrolyte. Figure 6 shows the CV curves of various Ni/NCO/RGO electrodes at scan rates of 2 to 400 mV/s with potential windows ranging from 0 to 0.45 V, respectively. From these figures, it is seen that for all the electrodes the integrated area under the curve and hence the current density increases with increasing scan rates, while retaining the same non-rectangular shapes. The non-rectangular shape of the CV curve represents the faradaic nature of the electrodes. Further, in the positive potential range, the CV curves of all electrodes reveal the existence of redox peaks which is attributed to the ongoing electrochemical redox reactions rising from the high presence of oxygen functional groups in the RGO nanosheets, which have high redox reactivity characteristics in the positive potential window [43,44]. Conversely, the redox peaks are almost diminished in the negative potential range of the CV curves of all electrodes, due to a kinetic irreversible process going on in the electrode material [45]. Thus, the presence of the oxidation peak in the forward scan and subsequent nonappearance of the reduction peak in the reverse scan indicates the occurrence of the irreversible reaction in the present case. Such irreversibility may be attributed to the considerably shorter lifetimes of the oxidized forms of the majority of known redox species than the voltammetry acquisition times, but further examination and experiments are required to find the exact mechanism of irreversibility [46]. The greater current response seen in the positive potential window compared to the negative potential range is because electrode materials are inclined to work in the positive potential range based on the high redox reactivity characteristics, as is shown in the CV test results obtained. The oxidation peak in the CV of the observed irreversible process shows gradual shifts to a higher potential value with an increase of scan rate due to the overpotential [47]. Moreover, it is important to mention that the shape of the CV curves for the electrode remains the same at all scan rates, revealing the outstanding electrical conductivity and decent rate competency of the electrode material.

**Figure 7:**
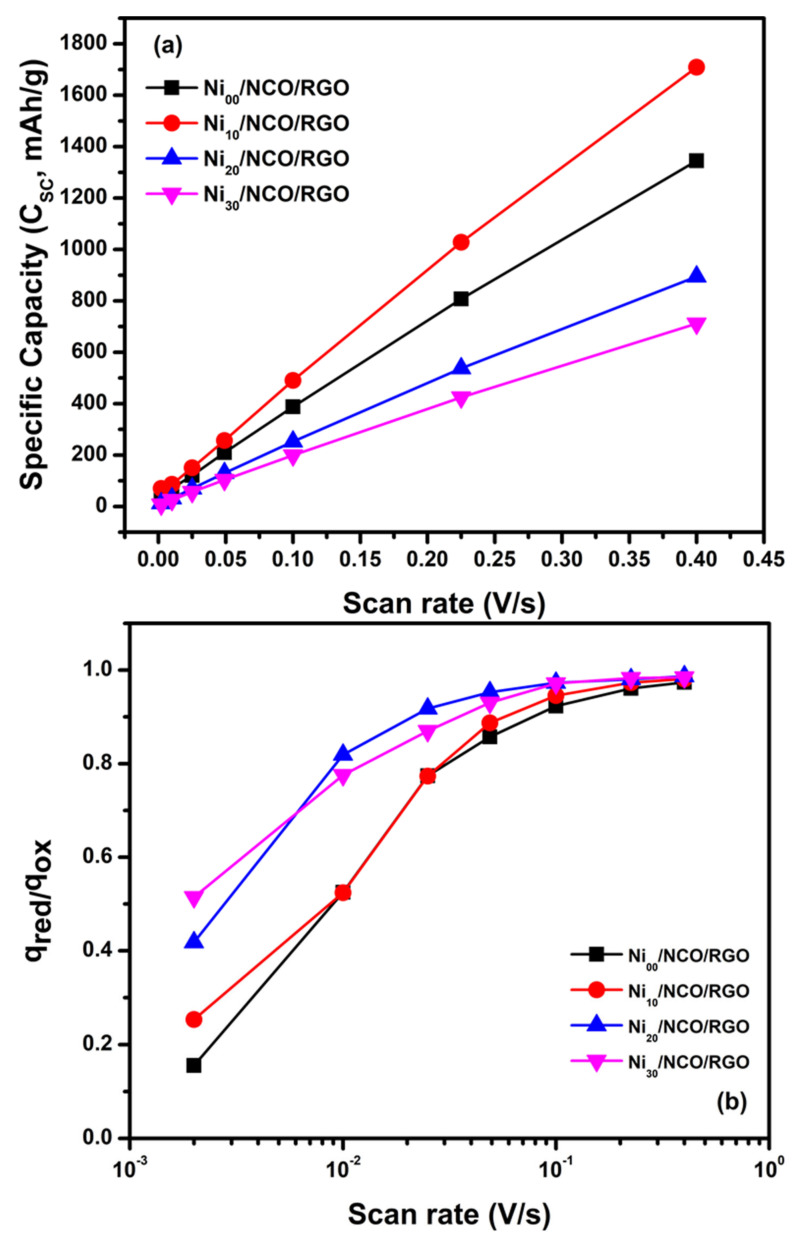
(**a**) Specific capacity and (**b**) coulombic efficiency q_red_/q_ox_ for various Ni/NCO/RGO electrodes at various scan rates.

In the present case, the electrochemical signature, e.g., CV curve, is not truly pseudocapacitive, but it is close to that of battery material. Therefore, instead of specific capacitance (*C*_sp_, F/g), the term specific capacity (*C*_sc_, mAh/g) is used to define the capability of the material as reported in much of the recent literature [48,49,50,51,52,53]. Herein, the specific capacity values for all electrodes were calculated by taking integration over the oxidation scan and these values are presented for the applied scan rate for each electrode in Figure 7a. Moreover, it is clear from Figure 6 that q_ox_ appears systematically higher than q_red_ and this suggests the occurrence of an irreversible reaction. Thus, to evaluate the irreversibility of the redox processes, the charge was further calculated by integrating the oxidation as well as reduction scans of CV curves at various scan rates for each electrode. Comparing the two processes, it is seen that the coulombic efficiency q_red_/q_ox_ value ranges between 0.15 to 0.98 at various applied scan rates for all electrodes (Figure 7b), and the efficiency is low at low scan rates and increases as the scan rate increases from 2 mV/s to 400 mV/s. The less than one value of observed coulombic efficiency illustrates the irreversibility of redox processes for all electrodes. Further, from Figure 7a, it is seen that the specific capacity of all electrodes increases almost linearly with an increase in scan rate. At all scan rates, the specific capacity is observed to be highest for the Ni_10_/NCO/RGO electrode in comparison to the other electrodes due to its highly porous nanostructure and the large effective surface area. The better surface area commendably improves the charge storage mechanism, offers abundant redox-active sites, and allows enhanced penetration of electrolyte ions. 

To demonstrate the importance of the NCO/RGO hybrid nanostructures, the CV curves of the Ni_10_/NCO electrode were also obtained at various scan rates, and the results are shown in Figure S4. Notably, the current of the Ni_10_/NCO/RGO electrode is much higher than that of the Ni_10_/NCO electrode, and the CV integrated area from the pristine NCO electrode is negligibly small compared with the RGO loaded NCO electrode. The seemingly smaller CV integrated area for the Ni_10_/NCO electrode indicates that its specific capacity is considerably smaller than that of the Ni_10_/NCO/RGO electrode. Figure S5 shows the variation of *C*_sc_ values calculated from the CV curves of the Ni_10_/NCO electrode at various applied scan rates. Evidently, at 400 mV/s scan rate, the integral area under the CV curve of Ni_10_/NCO/RGO electrode gives a specific capacity of 1710 mAh/g, and is much lesser for the Ni_10_/NCO electrode. By comparison, it is clear that even at a high scan rate of 400 mV/s, the specific capacitance of the Ni_10_/NCO/RGO electrode is about 90 times higher than that of the Ni_10_/NCO electrode. This larger *C*_sc_ could be due to both the unique hybrid nanostructure and the synergetic effects from the RGO nanoflakes and the NCO hollow microspheres. The RGO nanoflakes are well grown and distributed on the surface of the NCO microspheres, forming additional porous surface morphology. This offers a higher surface area and more active sites for the rapid intercalation and deintercalation of cations (K^+^) [54]. The electrochemical characterization of the bare Ni_10_ wire electrode was also carried out by obtaining the CV curves at various scan rates under 0–0.5 V potential window and hence calculating the respective specific capacity values as shown in Figure S6a,b. It confirms that the contribution made by the Ni wire current collector to the charge storage performance of the Ni_10_/NCO/RGO electrode is insignificant and the active materials’ hybrid nanostructure contributes to most of the electrode’s charge storage capacity. 

**Figure 8:**
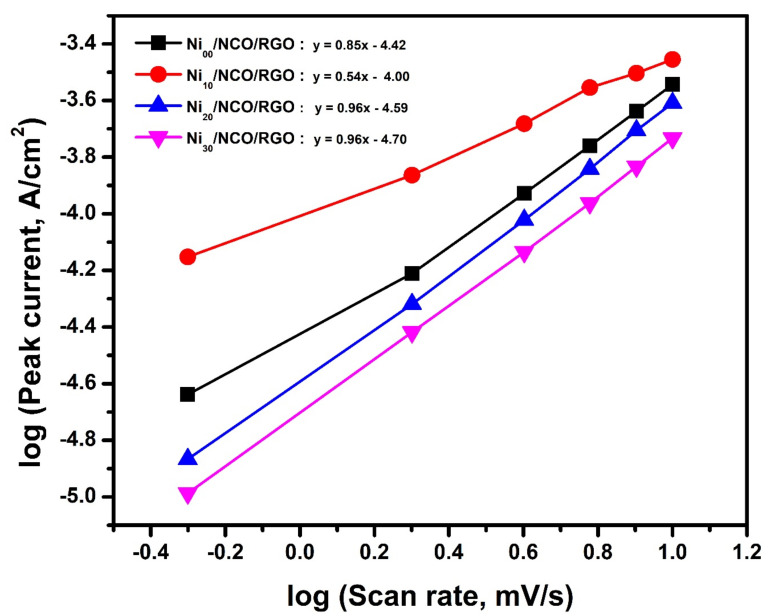
Dependence of the peak current on the scan rate (*υ*) for various Ni/NCO/RGO electrodes.

Moreover, to determine the charge transfer mechanism, the dependence of the peak current of the CV curve on the respective applied scan rate (*υ*) can be applied through the relation *i* = *aυ^b^*, where *a* and *b* are variable parameters. Accordingly, the observation of a value of 1.0 for *b* characterizes that the charge storage mechanism is mainly a surface-controlled process or capacitive process; while 0.5 specifies that the charge storage mechanism is a total diffusion-controlled process [55,56]. For Ni_00_/NCO/RGO, Ni_10_/NCO/RGO, Ni_20_/NCO/RGO, and Ni_30_/NCO/RGO electrodes, the *b* value was observed to be 0.85, 0.54, 0.96, and 0.96, respectively, from the slope in Figure 8, which directs a diffusion-controlled process related to the faradaic volume processes (battery-type) for the Ni_10_/NCO/RGO electrode and capacitive processes for other electrodes. This capacitive contribution is related to capacitive (EDLC) and pseudocapacitive (faradaic, capacitive-like signature) [48]. 

**Figure 9:**
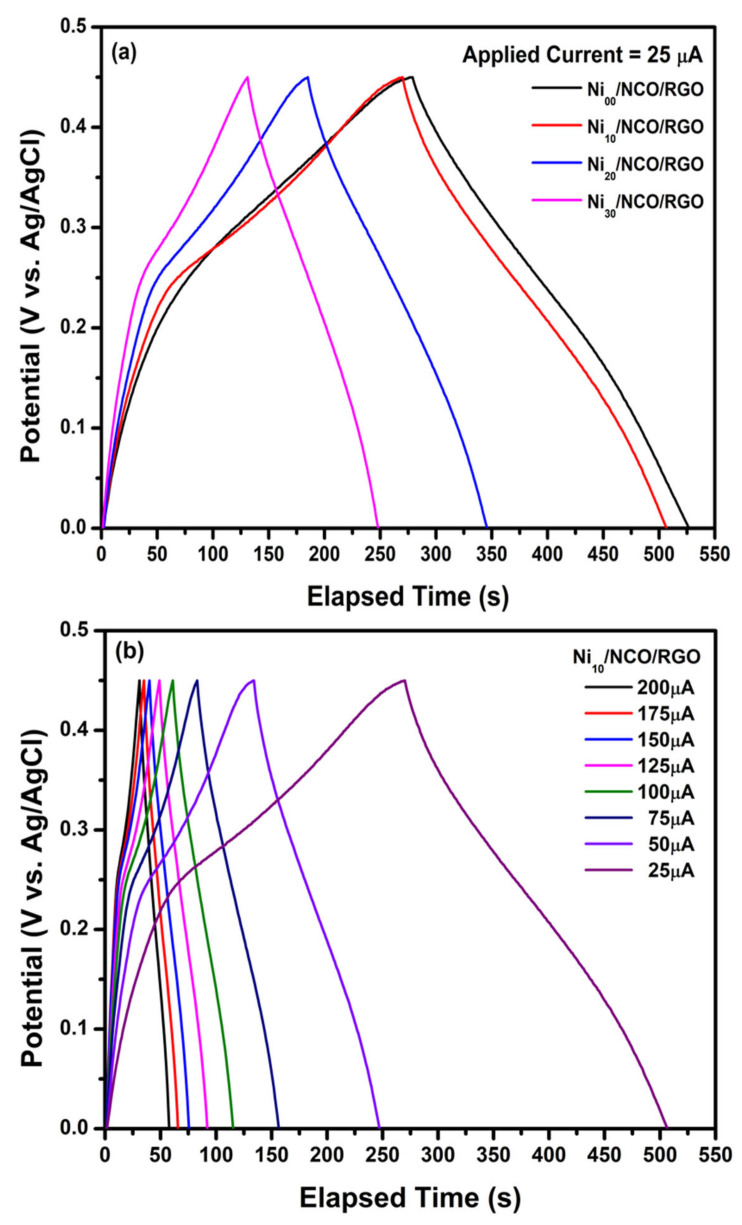
Galvanostatic charge/discharge (GCD) curves of (**a**) various Ni/NCO/RGO electrodes at applied currents of 25 μA and (**b**) Ni_10_/NCO/RGO electrode at various applied currents.

Figure 9a shows GCD curves of the various Ni/NCO/RGO electrodes within a potential window ranging from 0 to 0.45 V at an applied current of 25 μA; while Figure 9b shows the GCD plot of Ni_10_/NCO/RGO electrode at various applied currents of ranging from 25 μA to 200 μA. The triangular shape and symmetry of the GCD curves within the measured potential range confirms the excellent electrochemical reversibility of all electrodes. Besides, there are weak plateaus apparent at around 0.25 V, which follow the above-discussed CV results. Further, the nearly-linear galvanostatic discharge curves of all electrodes display a very good propagation of charges across the Ni/NCO/RGO electrode and KOH electrolyte. This also shows that a conductive network is created with proper pore channels through RGO nanoflakes in the hybrid nanostructure of the electrode, so offering a short electron and ion diffusion path for charge and electrolyte ions transfer between KOH electrolyte and the Ni/NCO/RGO electrode [57,58]. From Figure 9b, it is observed that the discharge time and hence the capacitance was constantly decreased along with an increase in current density for the Ni_10_/NCO/RGO electrode, which may be as a result of the low penetration of the ions into the inner region of pores of hybrid nanostructures due to fast potential variations. It is further clearly seen from Figure 9a that the discharge time has decreased with an increase in Ni wire etching time and the discharge time at an applied current of 25 μA are observed to be 248, 237, 161, and 117 s for Ni_00_/NCO/RGO, Ni_10_/NCO/RGO, Ni_20_/NCO/RGO, and Ni_30_/NCO/RGO electrodes, respectively. By using these discharge time values, the specific capacity values of electrodes were estimated, and the Ni_10_/NCO/RGO, Ni_20_/NCO/RGO, and Ni_30_/NCO/RGO electrodes exhibit 6.20, 5.93, 4.03, and 2.93 C/g specific capacities, respectively, at 25 μA. The GCD measurements showed that the specific capacity was at a maximum at the lowest applied current, and decreased with an increase in applied current. At 200 μA, the *Qs* value of the Ni_10_/NCO/RGO electrode is observed to be 5.40 C/g. This result was well illustrated in the charge-discharge plots in Figure 9a where the *Qs* value decreased for the Ni_10_/NCO/RGO electrode with ~91% rate capability. Furthermore, it is seen that the resultant GCD plots of all electrodes appear capacitive as a result of the combination of a capacitive electrode (triangular shape) and a Faradaic electrode (very weak plateau shape). 

In this work, specific capacitance values were also evaluated along with specific capacity. Since the diameter of the electrode was observed to decrease with Ni wire etching treatment, the volumetric (*C_v_*), as well as areal (*C*_A_) specific capacitance evaluated by using the above-observed discharge times, is the highest for the Ni_10_/NCO/RGO electrode. At 25 μA, the *C_v_* and *C*_A_ values possessed by the Ni_10_/NCO/RGO electrode are observed to be 2.89 F/cm^3^ and 27.7 mF/cm^2^, respectively. The *C_v_* values were further evaluated by considering only the active material volume and it was found that the Ni_10_/NCO/RGO electrode possesses a *C_v_* value of 253 F/cm^3^ at 25 μA. On account of its highly porous hybrid nanostructure and hence the high surface area, the electrode exhibited substantially-high specific capacitance (*C_v_* = 2.64 F/cm^3^ and *C*_A_ = 25.3 mF/cm^2^) even at a high applied current of 200 μA. Moreover, it was found that the Ni_10_/NCO/RGO electrode exhibits a wide potential window of 0.45 V compared to Ni_10_/NCO (Figure S7), which helps to improve the energy and power densities of the supercapacitor. The GCD measurements of the bare Ni_10_ wire electrode were at different applied currents (Figure S8) and the contribution made by the Ni wire current collector to the charge storage performance of the Ni_10_/NCO/RGO electrode were observed to be very negligible.

**Figure 10:**
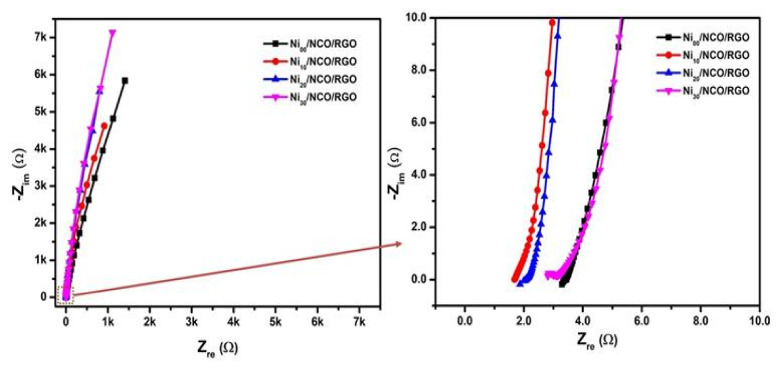
Electrochemical impedance spectroscopy (EIS) study (Nyquist plot) of various Ni/NCO/RGO electrodes at 100 kHz–0.01 Hz under 0.01 V.

Comparisons of electrochemical performances between various Ni/NCO/RGO electrodes were further carried out via EIS measurement. The Nyquist plots in Figure 10 reveal a small semicircle in the high-frequency region that relates to the charge transfer resistance (*R*_ct_), which is sequentially associated with the faradaic processes, and a linear part in the low-frequency region, signifying the capacitive behavior. As shown in the figure, the Ni_10_/NCO/RGO electrode expresses a relatively smaller semicircle at high frequencies than that of other electrodes, specifying reduced charge transfer resistance exhibited by the Ni_10_/NCO/RGO electrode. Since the effects of mass transfer of the OH^-^ ions from KOH solution on the impedance can be ignored at high frequency, the first data point in the high-frequency region displays the electrode/electrolyte interface’s initial resistance [59]. Thus, from the high-frequency region Nyquist plot, the initial resistances of Ni_00_/NCO/RGO, Ni_10_/NCO/RGO, Ni_20_/NCO/RGO, and Ni_30_/NCO/RGO electrodes were measured as 3.29, 1.68, 1.86, and 2.81 Ω, respectively. The obtained initial equivalent resistance of the Ni_10_/NCO/RGO electrode is lower than the initial resistances obtained for NiCo_2_O4 and NiCo_2_O_4_/MnO_2_ hierarchical nanostructure electrodes in our previous research work [60]. Moreover, the initial equivalent resistance of the Ni_10_/NCO/RGO electrode is comparable with that of 3D-nickel cobalt-layered double hydroxide onto the 3D-nickel wire (NiCo LDH/3D-Ni) and the Co(OH)_2_@Ni(OH)_2_/3D-Ni/NW electrode reported by Kang et al. [61] and Sharifi et al. [62], respectively. The initial resistance of the Ni_10_/NCO/RGO electrode is much smaller than the Ni/NCO electrode (Figure S9), which shows the importance of RGO in lowering the resistance of RGO. Besides, the line slope of the Ni_10_/NCO/RGO electrode is greater than that of other electrodes, which indicates the enhanced diffusion of electrolyte ions from the KOH solution to the electrode surface. Moreover, compared to other electrodes the Ni_10_/NCO/RGO electrode exhibits a relatively shorter linear portion in the plot attributable to its better capacitive behavior. Therefore, these results reveal that the Ni_10_/NCO/RGO electrode has the finest capacitive performance compared to the other fabricated electrodes.

### 3.3. The Electrochemical Characteristics of Ni_10_/NCO/RGO Wire-Supercapacitor

**Figure 11:**
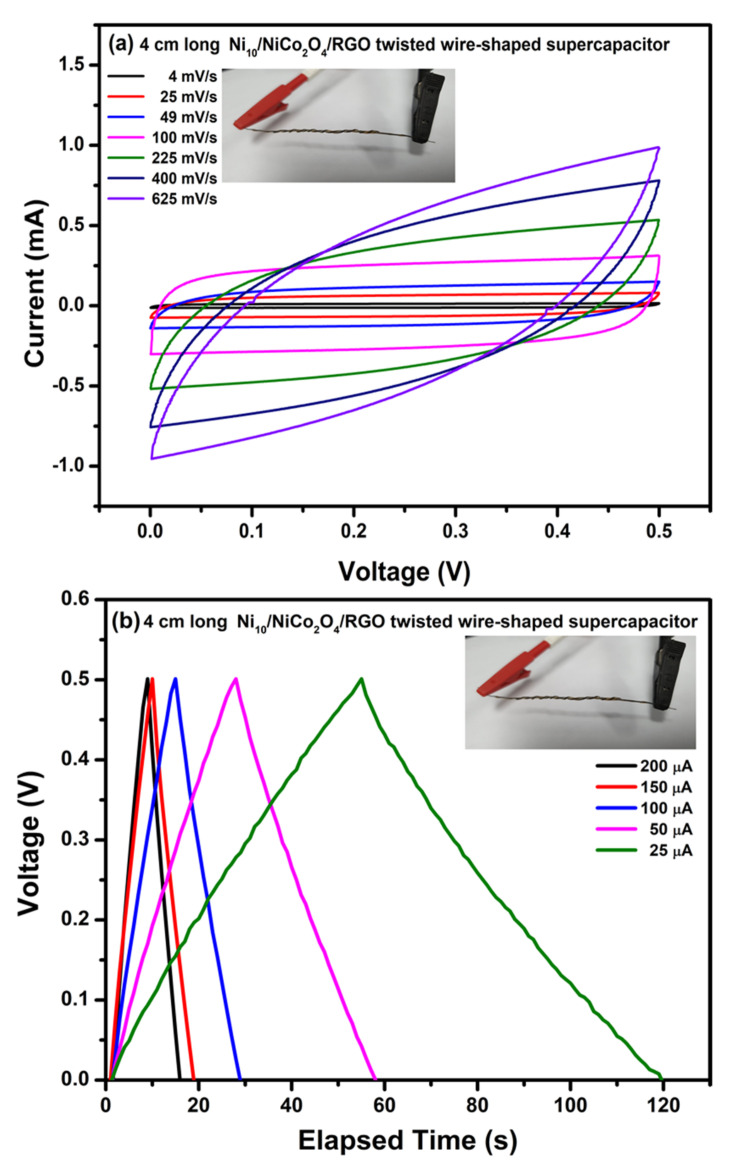
Electrochemical characterization of 4 cm long Ni_10_/NCO/RGO twisted WSC: (**a**) CV curves at various scan rates under 0–0.5 V potential window and (**b**) GCD curves at different applied currents.

**Figure 12:**
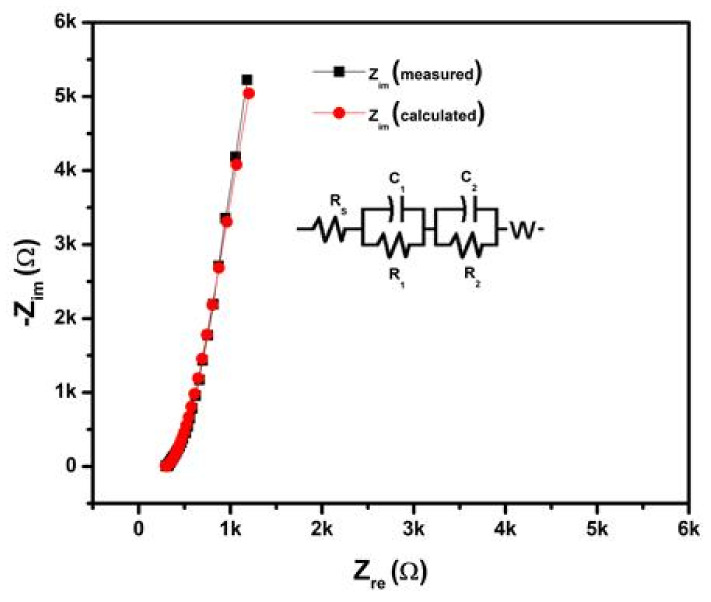
Electrochemical Impedance Spectroscopy (EIS) study (Nyquist plot) of 4 cm long Ni_10_/NCO/RGO twisted wire-shaped supercapacitor (WSC) at 100 kHz–0.01 Hz under 0.01 V (Inset shows the electrochemical equivalent circuit).

To further evaluate the practical applicability of optimum electrodes, we constructed the symmetric WSC with Ni_10_/NCO/RGO electrodes and PVA–KOH as the gel electrolyte as discussed earlier in the experimental section. Figure 11a exhibits the CV curves of the prepared 4 cm long Ni_10_/NCO/RGO twisted WSC device obtained at various scan rates with voltage windows ranging from 0 to 0.5 V. At the low scan rates, the CV voltammograms are virtually rectangular as well as symmetric. By increasing the scan rate, the CV curve begins to take a semi-rectangular (ellipsoidal) shape signifying the relative rise in the contribution of the pseudocapacitive mechanism. Figure 11b shows the GCD curves of the Ni/NCO/RGO WSC device within a voltage window of 0–0.5 V at various applied currents. The symmetric triangular-shaped galvanostatic charge/discharge curves once again manifest good capacitive behavior. The specific capacity of the fabricated WSC was evaluated from these GCD plots, and it is found to be 0.90 C/g and 0.78 C/g at an applied current of 25 A and 200 A, respectively. Thus, the WSC device shows a good rate capability of ~87%. Additionally, the volumetric and areal capacitances of the device were also calculated by using these GCD plots. At an applied current of 200 μA, the device exhibits volumetric and areal capacitances of 342 mF/cm^3^ and 3.11 mF/cm^2^, respectively. Further, compared with other capacitance values normally calculated from electrochemical tests based on low mass, length capacitance is a more substantial standard for WSEs. Therefore, the length-specific capacitance (*C*_L_) of the prepared 4 cm long wire-shaped supercapacitor was evaluated and is found to be 0.39 mF/cm at 200 μA. The obtained cell capacitance was compared with the previously published reports and it was found that the *C*_L_ value obtained in this work is much better than that obtained for MoO_3_ based serpentine-shaped wire supercapacitor, where Lee et al. have fabricated serpentine-shaped wire supercapacitors with different lengths from 3 to 15 cm and have reported a maximum of 0.37 mF/cm linear capacitance [63]. Similarly, the achieved *C*_L_ values are greater than the graphene/CNT composite fiber electrode-based supercapacitors with a length of 1.2 cm and a linear specific capacitance of 27.1 μF/cm [64]. The insets in Figure 11a,b show a photograph of the actual WSC device during electrochemical testing. Figure 12 displays the measured and fitted Nyquist plots of the WSC device, obtained at 0.01 V AC voltage within the frequency range from 100 kHz to 0.01 Hz. The plot was analyzed using ZSimpWin software as per the electrical equivalent circuit displayed in the inset of the figure. From the figure, an initial interface resistance of ~297 Ω was observed between the electrode and electrolyte at a high frequency which is larger than that of the single electrode. The electron transfer capability was also seen to be slightly decreased during the redox reaction as there is an apparent increase in the radius of the semicircular plot in the magnified view. These observations elucidate the decline in the electrochemical performance of the WSC device as compared to that of its single electrode, though the larger line slope and shorter liner portion in the low-frequency plot demonstrate a good capacitive behavior of the device with adequate ion diffusion. Further, the fitted equivalent circuit provides a significant understanding of the different capacitive contributions and charge transfer processes of WSC electrodes. The obtained equivalent circuit comprises ohmic resistance Rs signifying the combination of intrinsic resistance of current collector, material/substrate interface resistance, and the ionic resistance of the electrolyte [65,66,67]. The first parallel connection of R_1_ and C_1_ components characterizes the charge transfer in the surface layer of the electrode materials [68]; while the Warburg impedance (W) denotes the diffusion transport of electrolyte ions in the semi-infinite geometry [69]. The components in the second parallel connection, i.e., R_2_ and C_2,_ indicate the faradaic charge transfer resistance across the electrode/electrolyte interface and capacitance, respectively. The subsequently fitted parameters of the EIS Nyquist plot are given in Table 1 and the minor error accompanying the fitted data clearly shows that the model is appropriately matching with the experimental values. Finally, the important parameters such as energy and power densities of the obtained device were also evaluated and it is found that the as-fabricated WSC device demonstrates 10 μWh/cm^3^ energy density and 4.95 mW/cm^3^ power density at 200 μA. The energy and power densities for the WSC device at 200 μA were also calculated using the specific capacity, and their obtained values are 54 mWh/kg and 27 W/kg, respectively. 

**Figure 13:**
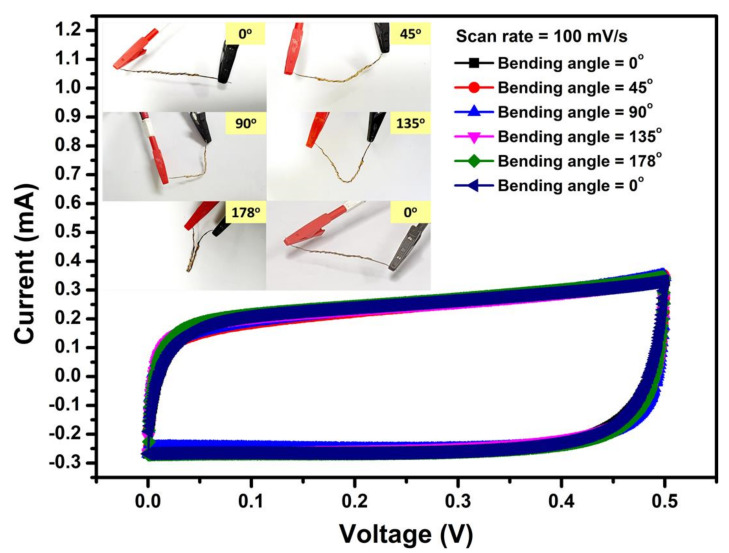
CV curves relating to various bending angles of Ni_10_/NCO/RGO twisted WSC at 100 mV/s scan rate. The inset shows the photographs of the flexible WSC with different bending angles.

The inset of Figure 13 shows photographs of a 4 cm long Ni_10_/NCO/RGO twisted WSC device in the conditions of its original state i.e., 0° bending angle, bending to a 45°, 90°, 135°, 178° and again to 0° bending angle. No cracks in the device were observed upon such bending deformations, showing superb flexibility of the as-fabricated WSC. To further confirm its high stability, CV curves of the as-fabricated WSC were compared before and after bending. Figure 13 shows CV curves at 100 mV/s scan rate corresponding to original, bending, and after bending states. The Ni_10_/NCO/RGO twisted WSC showed high flexibility and stability under the bending defamations, as it was illustrated that the areas of the curves corresponding to various bending angle deformations and after the bending state of the device were nearly identical to that measured under the original straight condition. The CV curves virtually overlap with each other, displaying good stability of the supercapacitor under different deformations.

**Figure 14:**
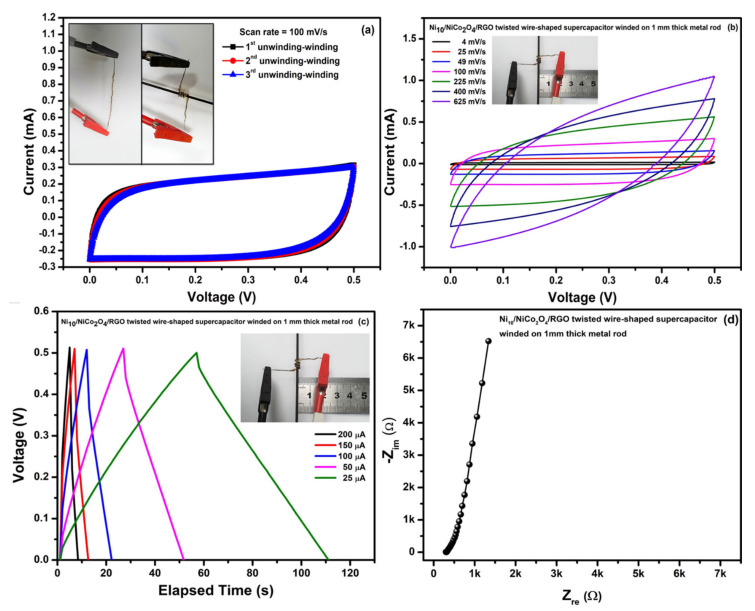
(**a**) CV curves relating to unwinding-winding sequences of Ni_10_/NCO/RGO twisted WSC at 100 mV/s scan rate, (**b**) CV curves for WSC in winded state at various scan rates, (**c**) GCD curves for WSC in winded state at different applied currents, and (**d**) EIS study (Nyquist plot) for WSC in winded state at 100 kHz–0.01 Hz under 0.01 V.

Moreover, the Ni_10_/NCO/RGO twisted WSC device winded for some laps on a metal rod with a diameter as small as 1 mm (inset of Figure 14a) and its CV curves were recorded after many unwinding (straight) and winding states. Figure 14a shows CV curves for 3 unwinding-winding sequences of the device at 100 mV/s scan rate; where CV is obtained for WSC when it is completely wound onto the metal rod. As can be seen from the figure, there are negligible changes in the CV curve shape and area under the curve. Furthermore, the CV at various scan rates (Figure 14b), GCD at various applied currents (Figure 14c) and EIS study (Figure 14d) were also performed in the last part, when the device had already been tested for obtaining CV curves with several bending and unwinding-winding sequences. The results of CV, GCD, and EIS measurements show insignificant variations between the winding state and the first state (Figure 11), suggesting that the WSC device retained steady energy storage characteristics and electron/charge diffusion kinetics. The electrochemical performance stability was attributed to the stable twisted configuration with adequate contact of the Ni_10_/NCO/RGO electrodes. 

**Figure 15:**
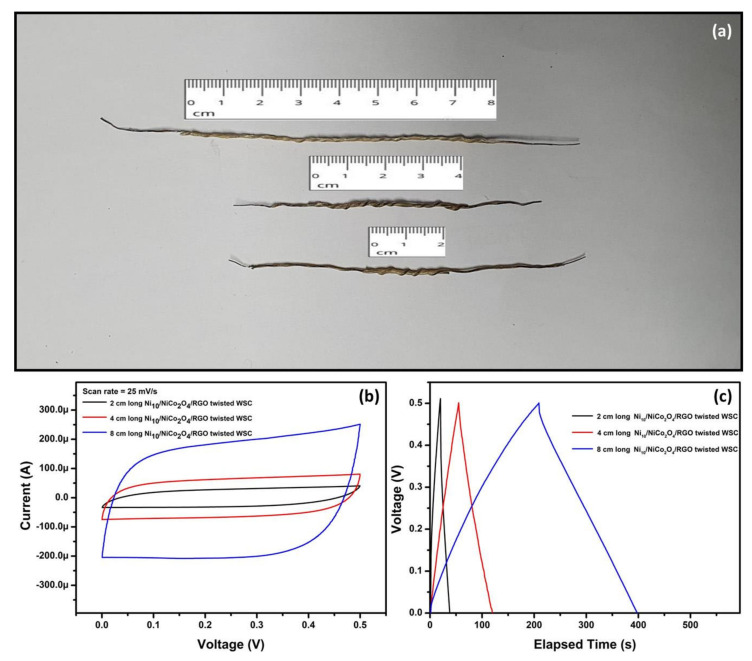
(**a**) Photograph of the fabricated Ni_10_/NCO/RGO WSCs with various lengths, (**b**) CV curves for various length Ni_10_/NCO/RGO WSCs at 25 mV/s scan rate under 0–0.5 V potential window, and (**c**) GCD curves obtained at applied currents of 25 μA for various length Ni_10_/NCO/RGO WSCs.

Finally, the Ni_10_/NCO/RGO twisted WSC device with different lengths of 2 and 8 cm was also fabricated and tested. The photograph of the Ni_10_/NCO/RGO WSCs with various lengths is shown in Figure 15a and their CV curves at 25 mV/s and GCD curves at 25 μA are shown in Figure 15b,c, respectively. The current in CV curves and discharge time in GCD curves increases almost linearly with the length. Therefore, the flexible wire-shaped supercapacitors fabricated in the present work can certainly be scaled up to achieve enhanced electrochemical characteristics concerning the need for wearable energy storage devices. 

## 4. Conclusions

In summary, we have successfully grown NiCo_2_O_4_/RGO hybrid nanostructures onto Ni wires as current collectors by using a combination of facile solvothermal and hydrothermal methods to obtain the wire electrodes, and hence fabricated the wire-shaped supercapacitor. The Ni wire surface was modified by the chemical etching process and the effect of etching on the structure, surface morphology, and electrochemical performance of the wire electrodes was thoroughly investigated. Results revealed that the microstructure, surface morphology, and electrochemical performance of the Ni/NiCo_2_O_4_/RGO electrodes were influenced significantly by Ni wire etching. The etching of the Ni wire current collector for 10 min led to the growth of hybrid nanostructures of spinel-NiCo_2_O_4_ hollow microspheres and RGO nanoflakes with the highest surface area and porosity. As a result, the diffusion-controlled process related to faradaic volume processes (battery type) contributed considerably over the surface-controlled process of the Ni_10_/NiCo_2_O_4_/RGO electrode compared to other electrodes, and hence the electrode shows the highest volumetric capacitance (2.64 F/cm^3^) and areal capacitance (25.3 mF/cm^2^) at 200 μA. The improved electrochemical performance of Ni_10_/NCO/RGO electrodes as compared to the Ni_10_/NCO electrode strongly shows an impact of synergistic effect between NiCo_2_O_4_ nanoparticles and RGO nanosheets. Further, a PVA/KOH gel electrolyte was coated onto such optimal Ni_10_/NCO/RGO electrodes and two electrodes assembled with in-twisted structure to obtain the wire-shaped supercapacitor, and its performance was demonstrated. The device exhibited reasonable energy and power densities of 10 μWh/cm^3^ (54 mWh/kg) and 4.95 mW/cm^3^ (27 W/kg), respectively. The as-fabricated WSC device is highly flexible and retains stable electrochemical characteristics under different deformations, such as bending and winding. The demonstration of the flexible wire-shaped supercapacitor fabrication with various lengths shows the scalability of the device fabrication to achieve enhanced electrochemical characteristics concerning the need for wearable energy storage devices.

## Figures and Tables

**Table 1:** EIS fit results of 4 cm long Ni_10_/NCO/RGO twisted WSC.

Element	Value
R_s_ (Ω)	295
C_1_ (μF)	2944
R_1_ (kΩ)	128.9
C_2_ (μF)	9.325
R_2_ (Ω)	19.01
W (mS∙s^1/2^)	3.333

## Data Availability

Not applicable.

## Supplementary Materials

The following are available online at https://www.mdpi.com/article/10.3390/nano11040852/s1, Figure S1. Photographs of the experimental set-up for (a) single electrode characterization by three-electrode configuration and (b) WSC characterization by two-electrode configuration. Figure S2. EDS spectra of various Ni/NCO/RGO electrodes with different substrate etching durations. Figure S3. (a) FE-SEM image, (b) EDS layer image, and the EDS mapping of (c) Ni (d) Co, (e) O, (f) C elements of Ni_10_/NCO/RGO electrode. Figure S4. CV curves of Ni/NCO electrode at various scan rates under 0–0.5 V potential window. Figure S5. Specific capacity of Ni/NCO electrode at various scan rates. Figure S6. Electrochemical characterization of bare Ni_10_ wire electrode: (a) CV curves at various scan rates under 0–0.5 V potential window and (b) specific capacitance of bare Ni_10_ wire current collector at various scan rates. Figure S7. GCD curves of Ni/NCO electrode at different applied currents. Figure S8. GCD curves of bare Ni_10_ wire electrode at different applied currents. Figure S9. EIS study (Nyquist plot) of the Ni/NCO electrode at 100 kHz–0.01 Hz under 0.01 V.
