# Supplementary material for: NiCo2O4/RGO Hybrid Nanostructures on Surface-Modified Ni Core for Flexible Wire-Shaped Supercapacitor
Source: Nanomaterials (Basel). 2021 Mar 26;11(4):852. doi: 10.3390/nano11040852 (PMC8066179; doi:10.3390/nano11040852)
Supplement: Supplementary file 1 [file nanomaterials-11-00852-s001.pdf]

Supplementary Materials

$NiCo_2O_4$/RGO hybrid nanostructures on surface-modified Ni core for flexible wire-shaped supercapacitor

Prashant Shivaji Shewale and Kwang-Seok Yun *

Department of Electronic Engineering, Sogang University, Seoul, 04107, Republic of Korea

prashantshewale11@gmail.com, * Corresponding Author: ksyun@sogang.ac.kr

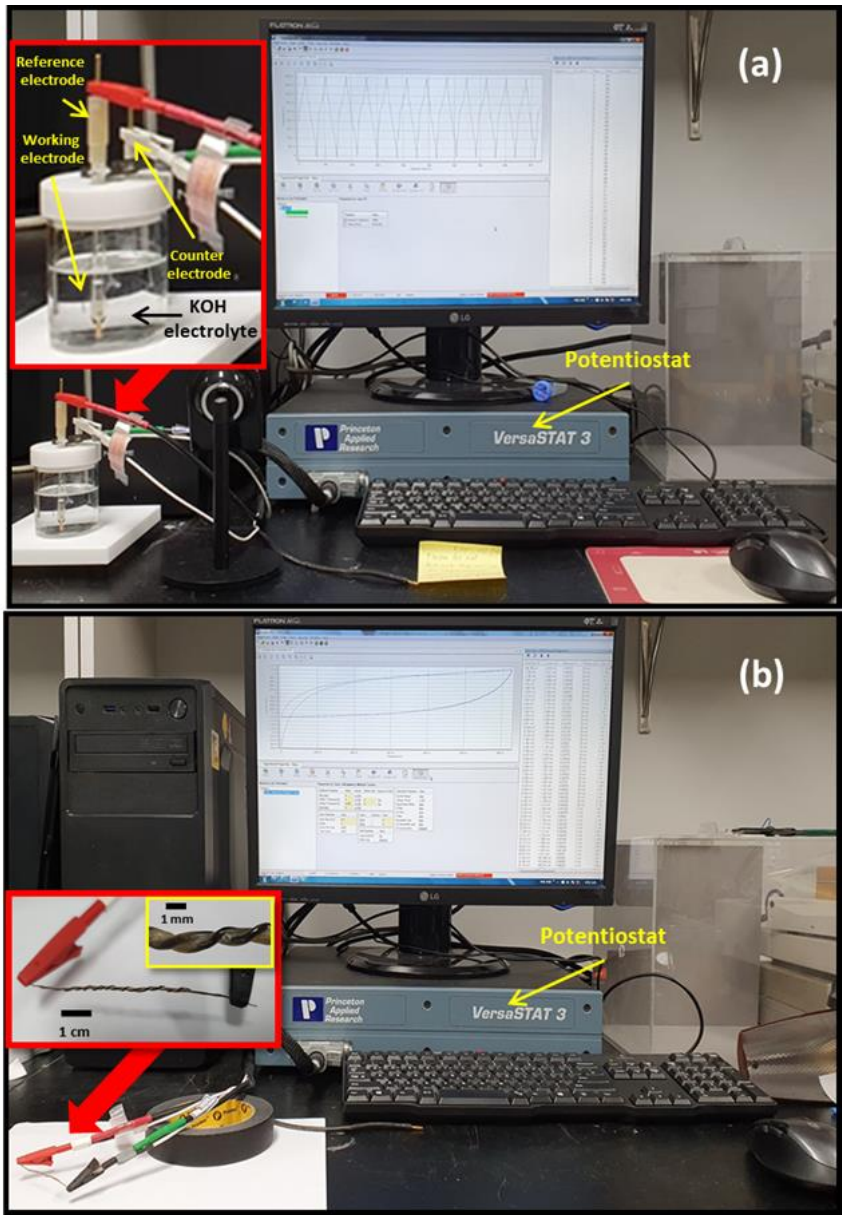

Fig. S1. Photographs of the experimental set-up for (a) single electrode characterization by the three-electrode configuration, (b) WSC characterization by the two-electrode configuration.

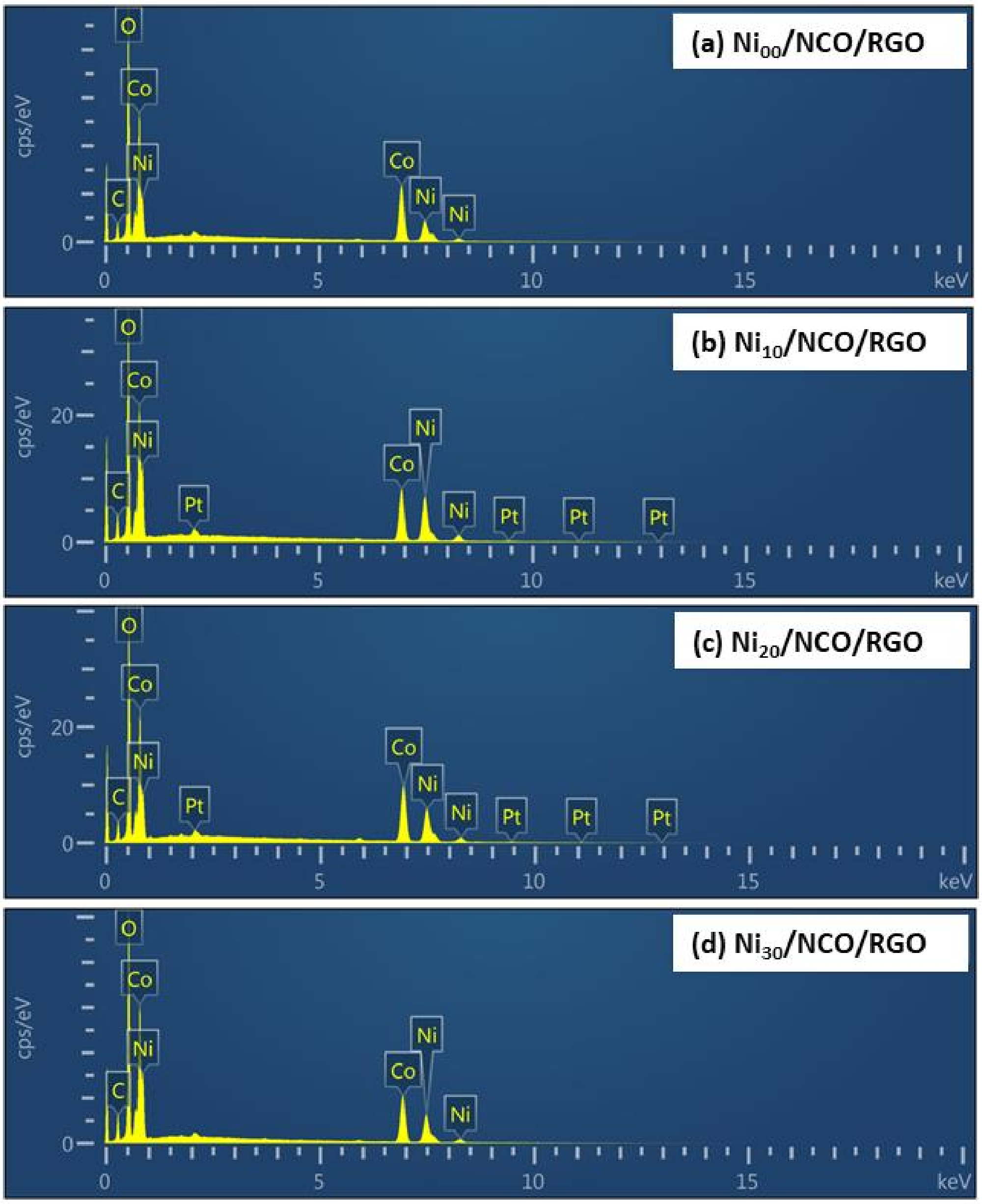

Fig. S2. EDS spectra of various Ni/NCO/RGO electrodes with different substrate etching durations.

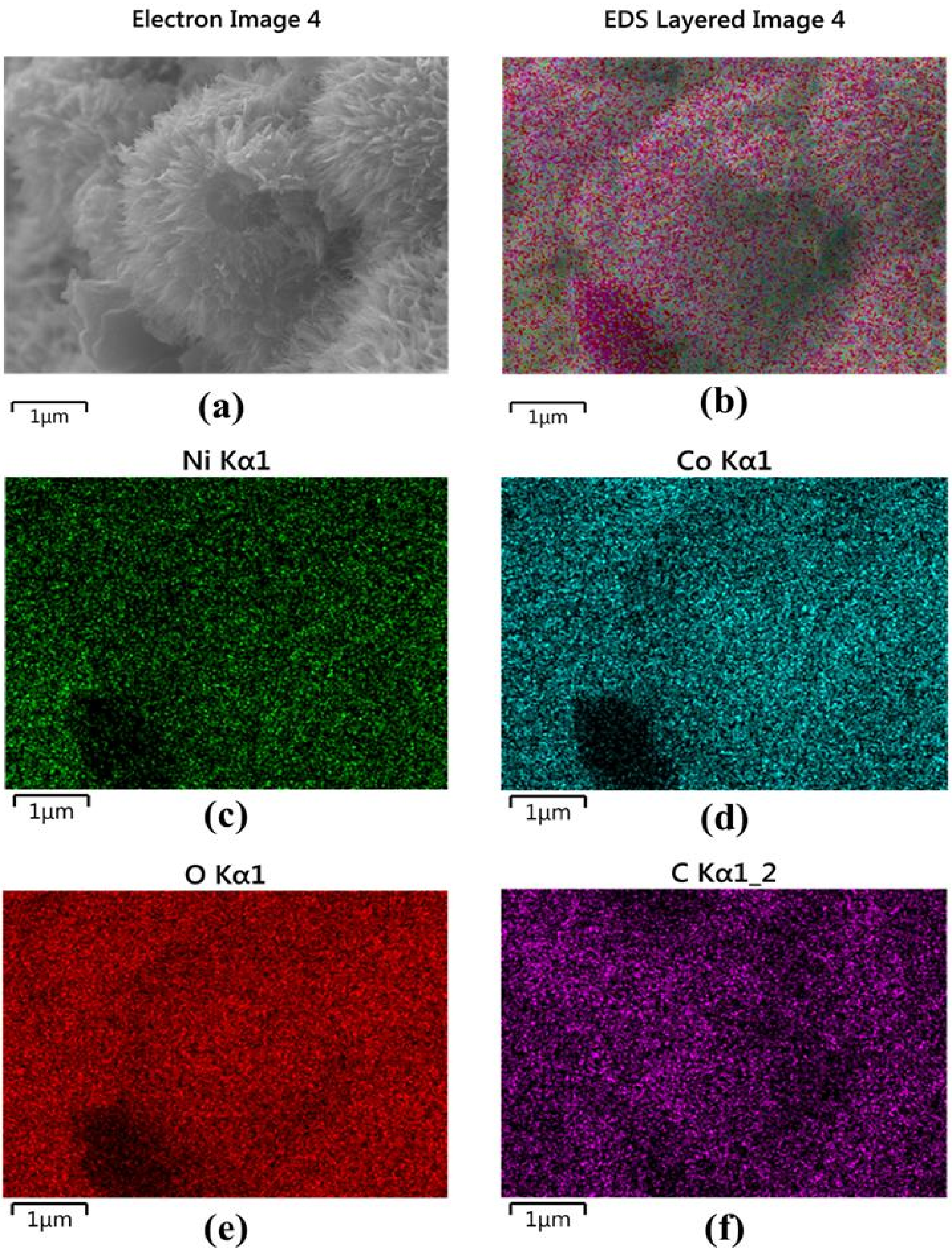

Fig. S3. (a) FE-SEM image, (b) EDS layer image, and the EDS mapping of (c) Ni (d) Co, (e) O, (f) C elements of Ni_{10}/NCO/RGO electrode.

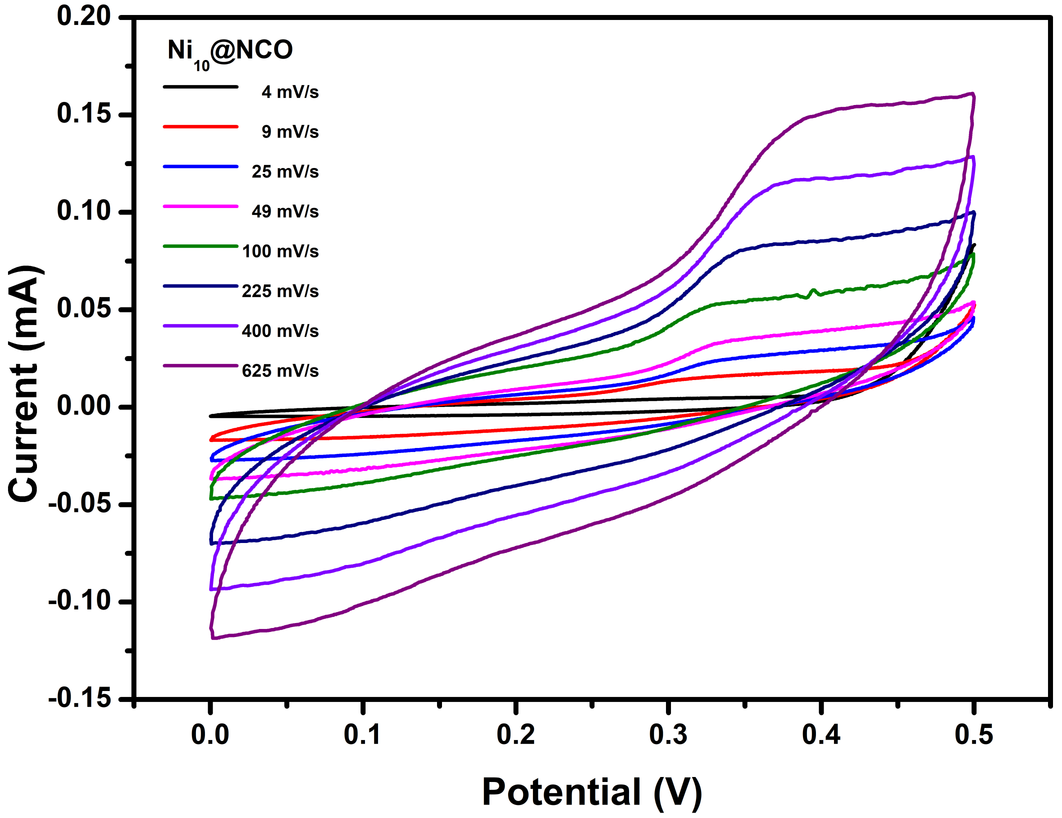

Fig. S4. CV curves of Ni/NCO electrode at various scan rates under 0-0.5 V potential window.

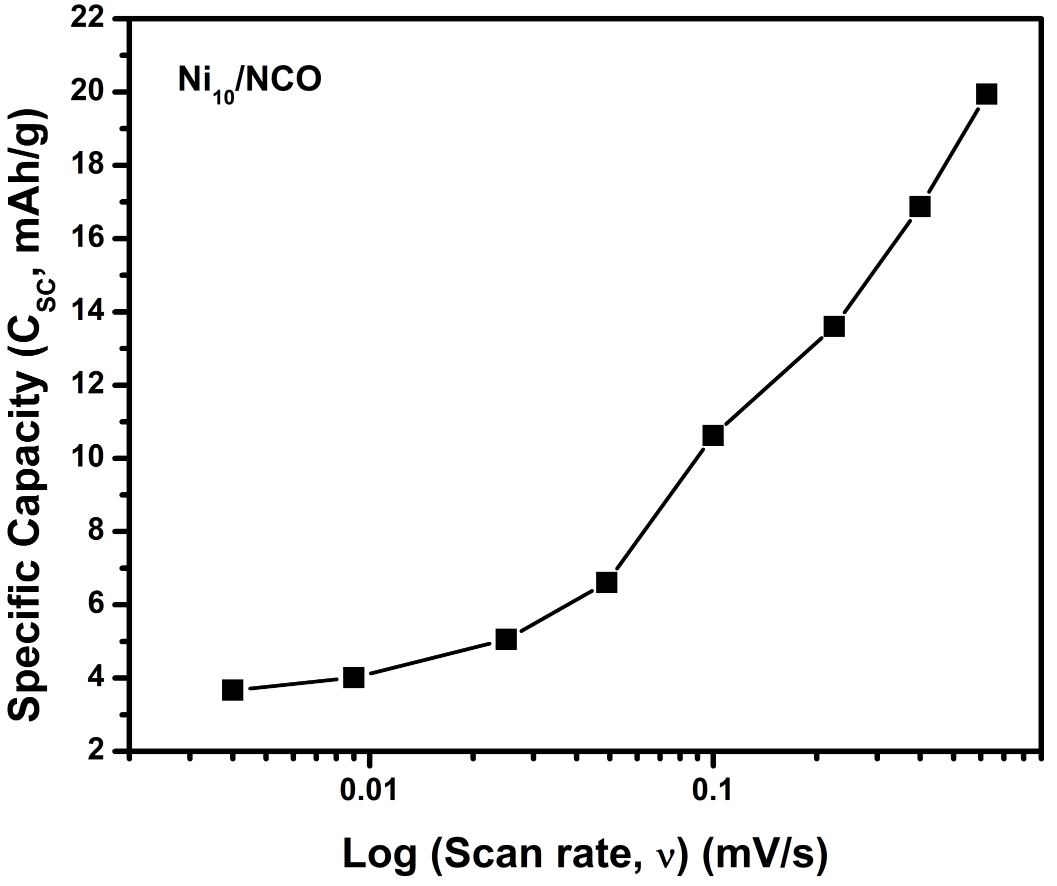

Fig. S5. Specific capacity of Ni/NCO electrode at various scan rates.

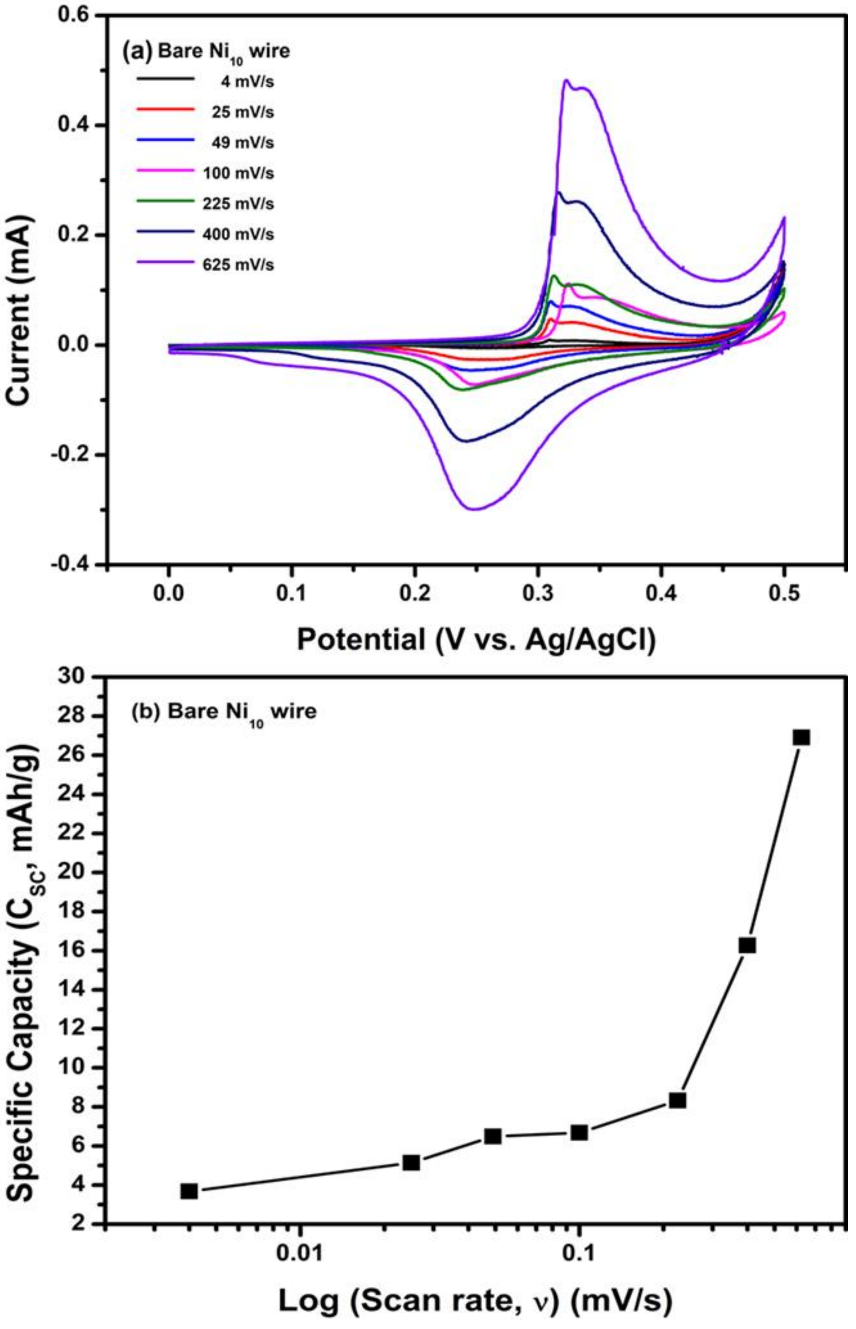

Fig. S6. Electrochemical characterization of bare Ni_{10} wire electrode: (a) CV curves at various scan rates under 0-0.5 V potential window, and (b) specific capacity of bare Ni_{10} wire electrode at various scan rates.

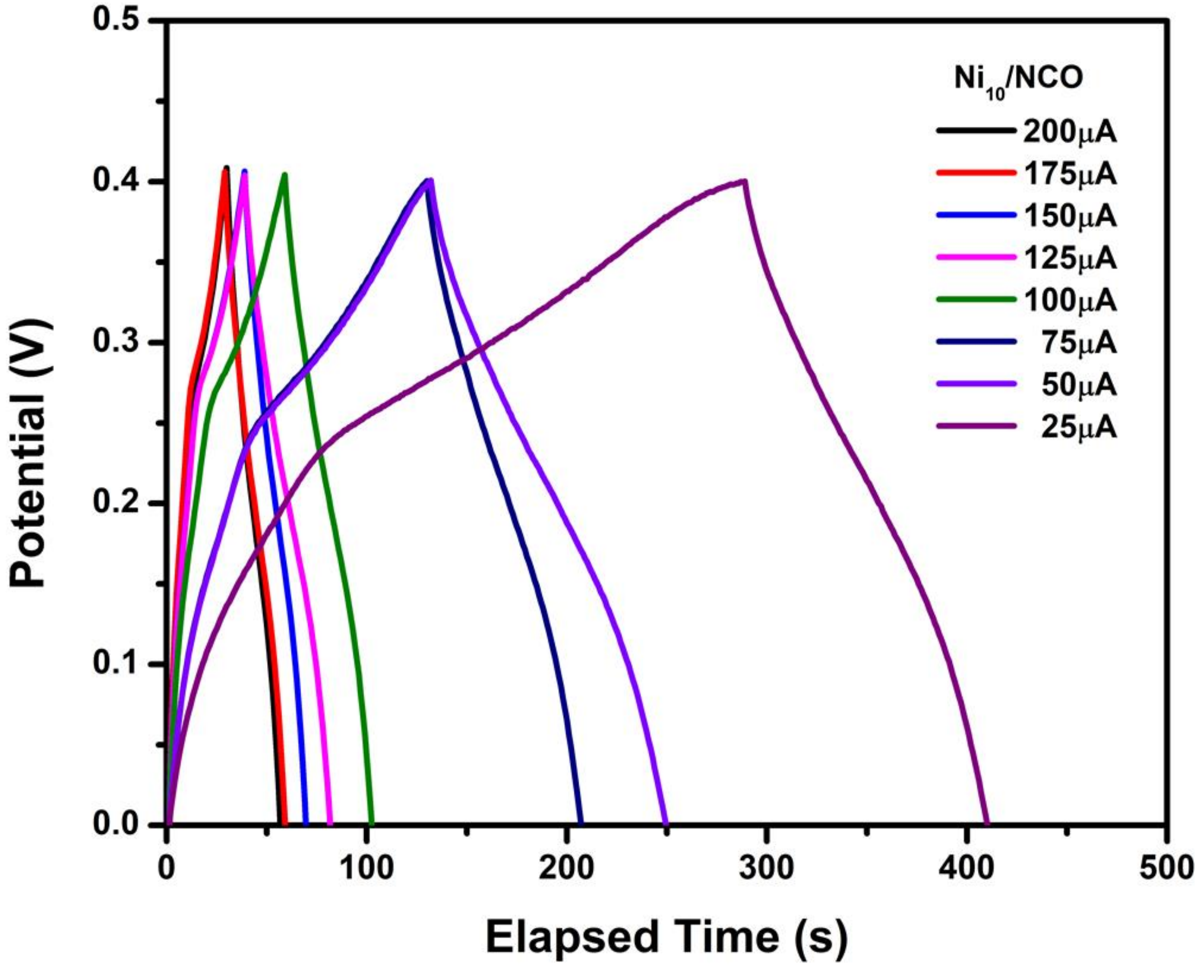

Fig. S7. GCD curves of Ni/NCO electrode at different applied currents.

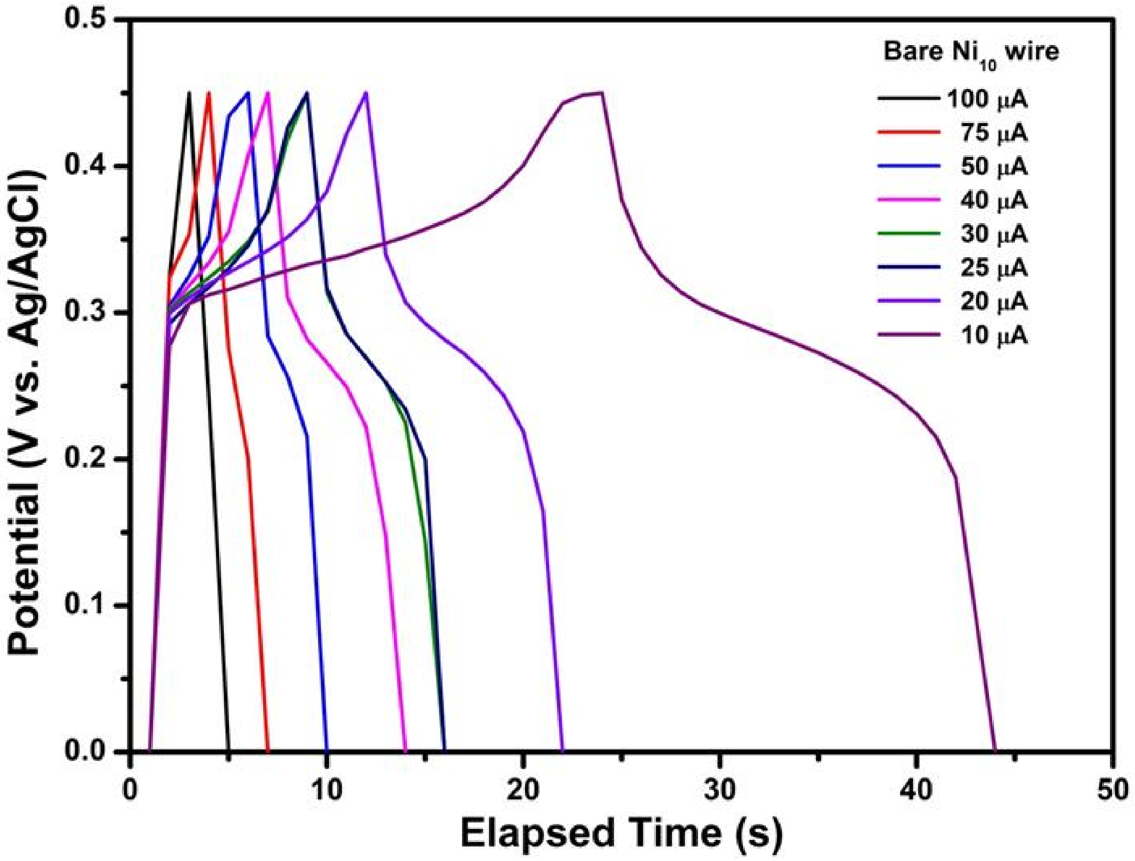

Fig. S8. GCD curves of bare Ni_{10} wire electrode at different applied currents.

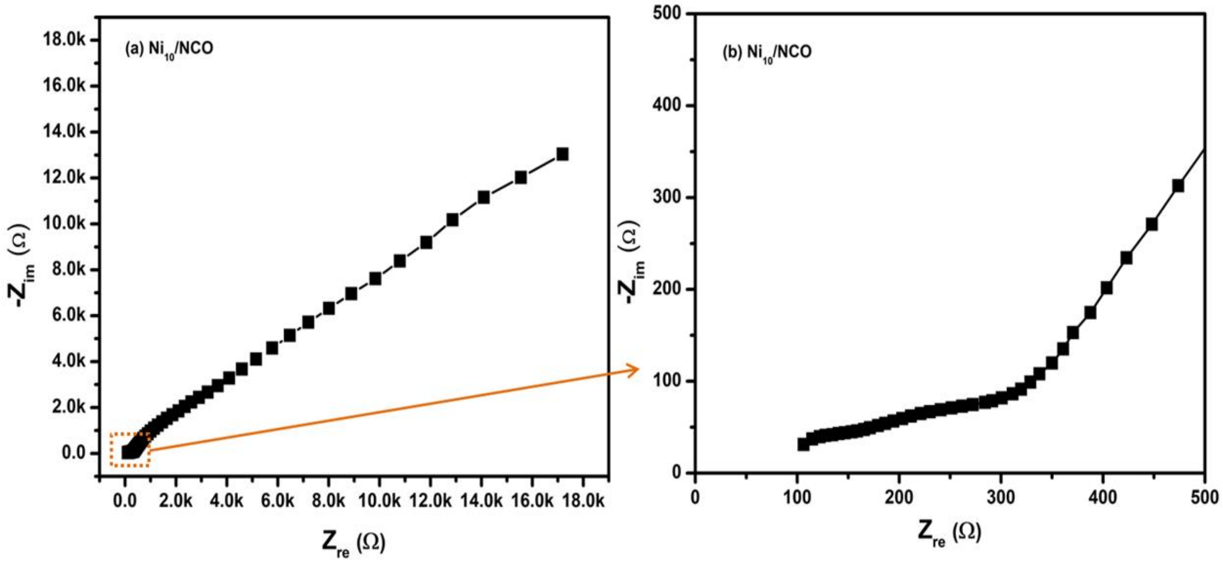

Fig. S9. EIS study (Nyquist plot) of the Ni/NCO electrode at 100 kHz-0.01 Hz under 0.01 V.
